# Radiology response in the emergency department during a mass casualty incident: a retrospective study of the two terrorist attacks on 22 July 2011 in Norway

**DOI:** 10.1007/s00330-016-4677-8

**Published:** 2016-12-12

**Authors:** Victoria Solveig Young, Heidi B. Eggesbø, Christine Gaarder, Pål Aksel Næss, Tone Enden

**Affiliations:** 1 Division of Radiology and Nuclear Medicine, Oslo University Hospital, PO Box 4950, Nydalen, N-0424 Oslo Norway; 2 Department of Traumatology, Oslo University Hospital, PO Box 4950, Nydalen, N-0424 Oslo Norway; 3 Department of Paediatric Surgery, Oslo University Hospital, PO Box 4950, Nydalen, N-0424 Oslo Norway; 4 Institute of Clinical Medicine, Faculty of Medicine, University of Oslo, PO Box 1171, Blindern, 0318 Oslo Norway

**Keywords:** Mass casualty incident, Emergency radiology, Radiology department, Disaster planning, Trauma

## Abstract

**Objectives:**

To describe the use of radiology in the emergency department (ED) in a trauma centre during a mass casualty incident, using a minimum acceptable care (MAC) strategy in which CT was restricted to potentially severe head injuries.

**Methods:**

We retrospectively studied the initial use of imaging on patients triaged to the trauma centre following the twin terrorist attacks in Norway on 22 July 2011.

**Results:**

Nine patients from the explosion and 15 from the shooting were included. Fourteen patients had an Injury Severity Score >15. During the first 15 h, 22/24 patients underwent imaging in the ED. All 15 gunshot patients had plain films taken in the ED, compared to three from the explosion. A CT was performed in 18/24 patients; ten of these were completed in the ED and included five non-head CTs, the latter representing deviations from the MAC strategy. No CT referrals were delayed or declined. Mobilisation of radiology personnel resulted in a tripling of the staff.

**Conclusions:**

Plain film and CT capacity was never exceeded despite deviations from the MAC strategy. An updated disaster management plan will require the radiologist to cancel non-head CTs performed in the ED until no additional MCI patients are expected.

***Key points*:**

*• Minimum acceptable care (MAC) should replace normal routines in mass casualty incidents.*

*• MAC implied reduced use of imaging in the emergency department (ED).*

*• CT in ED was restricted to suspected severe head injuries during MAC.*

*• The radiologist should cancel all non-head CTs in the ED during MAC.*

## Introduction

In a mass casualty incident (MCI), the capacity to provide optimal trauma care is unpredictable and will be challenged [1–4]. Consequently, normal routines are replaced by a minimum acceptable care (MAC) strategy aimed at rapid assessment and limited to lifesaving procedures using minimal resources, including imaging, followed by movement of patients to more definitive diagnostics and management [2, 3, 5]. Whether during an MCI or not, trauma imaging in the emergency department (ED) should include plain x-rays of chest and pelvis, and focused assessment with sonography for trauma (FAST) as a screening tool for free fluid [5]. When applying a MAC strategy, the use of computed tomography (CT) in the ED is restricted to the assessment of severe head injuries. This is different from the routine initial diagnostic work-up of potentially severely injured patients, with frequent use of head and body CT combined, in line with the Advanced Trauma Life Support (ATLS) radiological guidelines [1, 6, 7].

The two sequential terrorist attacks in Norway on the afternoon of 22 July 2011—a bomb explosion in central Oslo and a shooting spree at Utøya—caused (as of September 2016) the largest MCI in Norway since World War II, with more than 220 casualties [5, 8–10]. The medical literature assessing the use and logistics of radiological imaging during MCIs is scarce, and is based in part on table-top exercises and practice simulations [11, 12]. We are not aware of any reports focusing on radiology response in an MCI involving both a bomb explosion and a shooting incident.

The aim of this report was to describe the initial use of radiology resources for the patients triaged to the major trauma centre during this MCI.

## Materials and methods

The 25 victims who arrived at Oslo University Hospital Ullevål within the first 15 h following the two attacks were eligible for this retrospective study. The study was approved by the institutional data protection officer. Written consent was provided from all but one patient (or their next in kin), and thus 24 patients were included: nine from the bomb explosion and 15 from the shooting incident.

Oslo University Hospital Ullevål (OUHU) is the only regional trauma centre for the south-eastern health region in Norway, covering a population of 2.8 million. OUHU currently admits approximately 2000 trauma patients annually, of which more than 700 are severely injured with an Injury Severity Score (ISS) >15. The prehospital triage system in the Oslo area mandates potentially severely injured patients to be transported to OUHU, and prehospital personnel established effective triage near the locations of both incidents on 22 July 2011, following the same system [10].

The first terror attack at 15.25 was an explosion from a 950-kg fertilizer car bomb in the governmental quarter in Oslo. Eight people died on the scene, and ten were taken to the trauma centre with potentially life-threatening blast and shrapnel injuries. The first patient arrived in the ED 26 min after the explosion. Six more arrived within the next 19 min, and all nine from the government attack arrived within 2 h.

The second attack, shortly after 17.25, was a shooting spree at a political youth camp on Utøya, an island approximately 40 km northwest of Oslo. The weapons used were a handgun and a rifle with expanding ammunition. Sixty-eight people died on the scene, and more than 60 were injured. Of the 21 patients who were triaged and transported to OUHU, 15 arrived during the first 15 h. The first helicopter arrived at 19.57 with four patients. The last patient in this report arrived at 01.07 on 23 July.

Surgical leadership was divided between one surgeon responsible for triage and management of the ED logistics, and another supervising teamwork and treatment strategies beyond the ED. Anaesthesiology personnel were managed by a dedicated trauma anaesthesiologist. Up to six trauma teams were engaged in the ED simultaneously. The normal trauma team at OUHU consists of 8–15 people, including radiographer and radiologist. During an MCI, the trauma teams are reduced, but always include surgical trauma team leader, anaesthesiologist, nurse anaesthetist and examining surgeon, as well as ED nurse. Other personnel groups, including radiology personnel, will be used between the teams as needed.

Code red activation of the disaster management plan as elicited by the ED nurse coordinator included notification through the internal hospital calling system to the radiographer and the radiologist on call. They then contacted their respective leaders—one head radiologist and one head radiographer—in the abdominal and thoracic radiology unit by telephone. The two leaders contacted, in top-down sequence, all available radiology personnel in their unit and the units for neuro, orthopaedic, and vascular radiology, to come to or stay at the hospital and register in the RD. Radiology personnel and imaging resources were overviewed and organized directly from the RD by the two radiology leaders.

The main trauma room (on the ground floor) in the ED had three trauma bays, and was equipped with one portable ultrasound machine and one overhead x-ray gantry. Another four portable x-ray machines and one portable ultrasound machine were made available. One 64-slice CT scanner was located in the adjacent room. More imaging laboratories were located in three different floors in the radiology department (RD) in the connected neighbour building 90 m away, and included three 64-slice CT scanners, three x-ray machines, and two angiography suites. Operating rooms were located one floor up from the ED.

Unidentified patients were labelled by a standardised numbering and naming convention which was used for the patients’ electronic medical records, including the radiologic information system (RIS). This unique number was later merged in the records with the unique 11-digit personal identification number used in Norway. All radiological examinations except FAST were consecutively registered in the RIS and the picture archiving and communication system (PACS; both Siemens Healthcare, Erlangen, Germany) with this personal number, time, laboratory/department, and examination code.

The data for the study were obtained retrospectively from the patients’ medical records and the RIS. For the FAST results, some data were obtained from handwritten notes. Data on radiology staff were obtained from the hospital personnel system (www.gatsoft.no.Data). on the initial radiology response were primarily divided between the patients from the bombing (first attack) and the shooting spree (second attack), and secondly between imaging performed inside the ED, and after discharge to operation theatres or intensive care units, i.e. imaging in the RD or in the wards. The CT examinations were categorised as whole-body CT (WBCT), which included head, spine, chest, abdomen, and pelvis, as well as torso CT, head CT, and CT of the extremities. The time lapse of the radiology response was obtained from the RIS and broken down into 30-min periods.

## Results

A total of 24 victims from the two attacks were included in the study. All patients were triaged prehospital, and the trauma centre received no walk-in patients. Half were female, and the mean age was 26 years (standard deviation 15 years; Table **1**). The median ISS was 20, with injuries most commonly seen in the extremities (75%), followed by head and neck (58%), chest (50%), and abdomen (29%). In 15 patients (63%), injuries included more than one body region: six of nine from the bomb explosion and nine of 15 from the shooting incident.

**Table 1:** Patient characteristics

	Bomb explosion *n* = 9	Shooting incident *n* = 15	Total *n* = 24
Mean age (SD), years	37 (18.5)	18 (2.3)	26 (14.6)
Females (*n*)	4	8	12
Mean ISS (SD)	21 (18.3)	21 (15.9)	21 (16.4)
Median ISS (range)	18 (1–50)	20 (1–59)	20 (1–59)
ISS >15 (*n*)	5	9	14
Injured body region* (*n*)			
Head and neck	7	10	17
Chest	6	5	11
Abdomen and pelvis	3	5	8
Extremities	6	12	18
Radiological examinations			
Plain films	7	15	22
FAST	5	10	15
CT	6	12	18

*A patient may have >1 injured body region. *ISS* Injury Severity Score, *SD* standard deviation

**Fig. 1:**
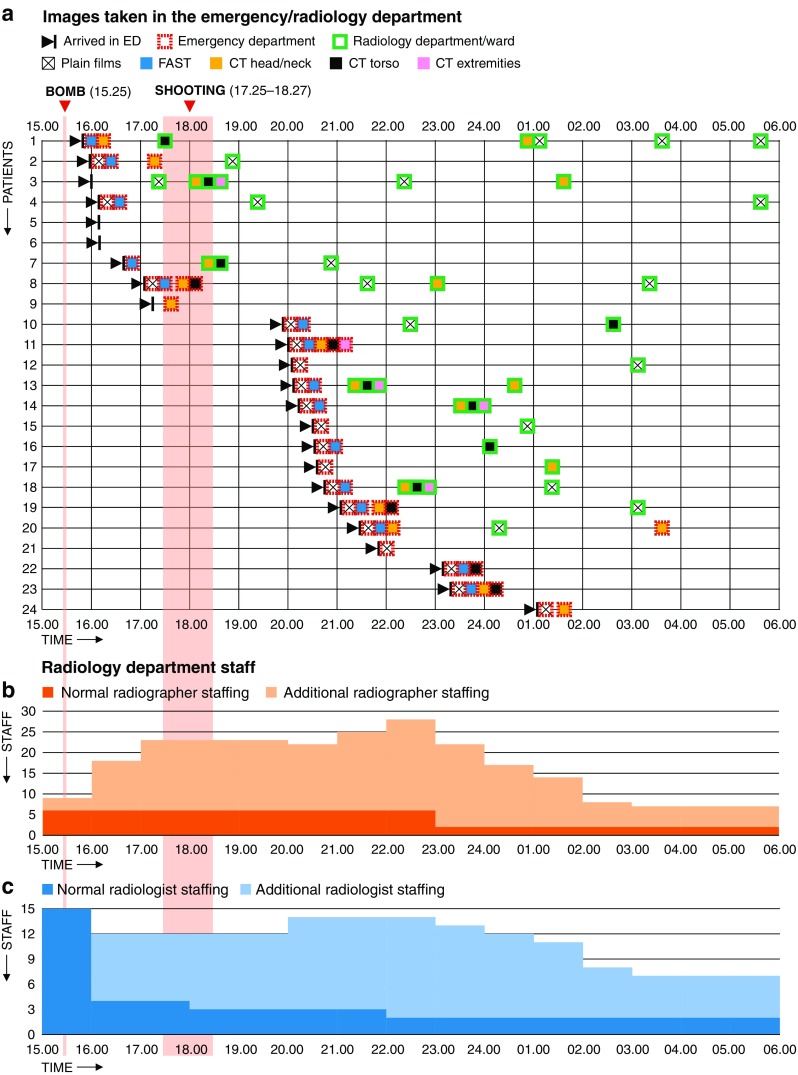
Initial imaging and radiology staff during the first 15 h. Patients 1–9 are from the bomb explosion and patients 10–24 are from the shooting incident. The *colour-coded boxes* indicate the various radiological examinations (see legends) in the emergency department (*red dotted frames*) and in the radiology department (*green frames*), respectively, and within time units of 30 min. The *lower two panels* show the number of radiographers and radiologists present per hour during the study period, respectively. Radiographers have their daily change of shift at 15.00, radiologists at 16.00

During the first 15 h, 22 patients (92%) underwent diagnostic imaging. No referrals for imaging were declined. Six of the nine patients from the bomb explosion underwent imaging in the ED, one more in the RD only. Two patients had scalp wounds only and were not subject to imaging in the ED. An overview of the radiology response is presented in Fig. **1**.

All 15 patients from the shooting incident had plain films (chest, pelvis, and/or extremities) in the ED, compared to only one in three patients from the bomb explosion. Later, 50% had plain films taken of the chest and/or extremities in the wards or the RD: six from the bomb explosion and six from the shooting incident. Chest x-rays constituted half of the plain films: 18 of the 42 (43%) plain films in the ED and 13 of the 23 (57%) taken in the wards or RD.

Five of nine patients from the bomb explosion and 10 of 15 from the shooting incident underwent FAST in the ED because of suspected abdominal or thoracic injury. Four of 15 FAST examinations were positive, three of which were in explosion patients. However, subsequent laparotomy was negative in two of them, revealing no intraabdominal injury. Due to the nature of the injuries (penetrating abdominal gunshot wounds), four of the patients from the shooting were directed to laparotomy with no FAST.

A total of 18 patients (75%) had a CT examination performed during the first 15 h after arrival in the ED (Fig. **1**): six from the bomb explosion and 12 from the shooting incident.

**Fig. 2:**
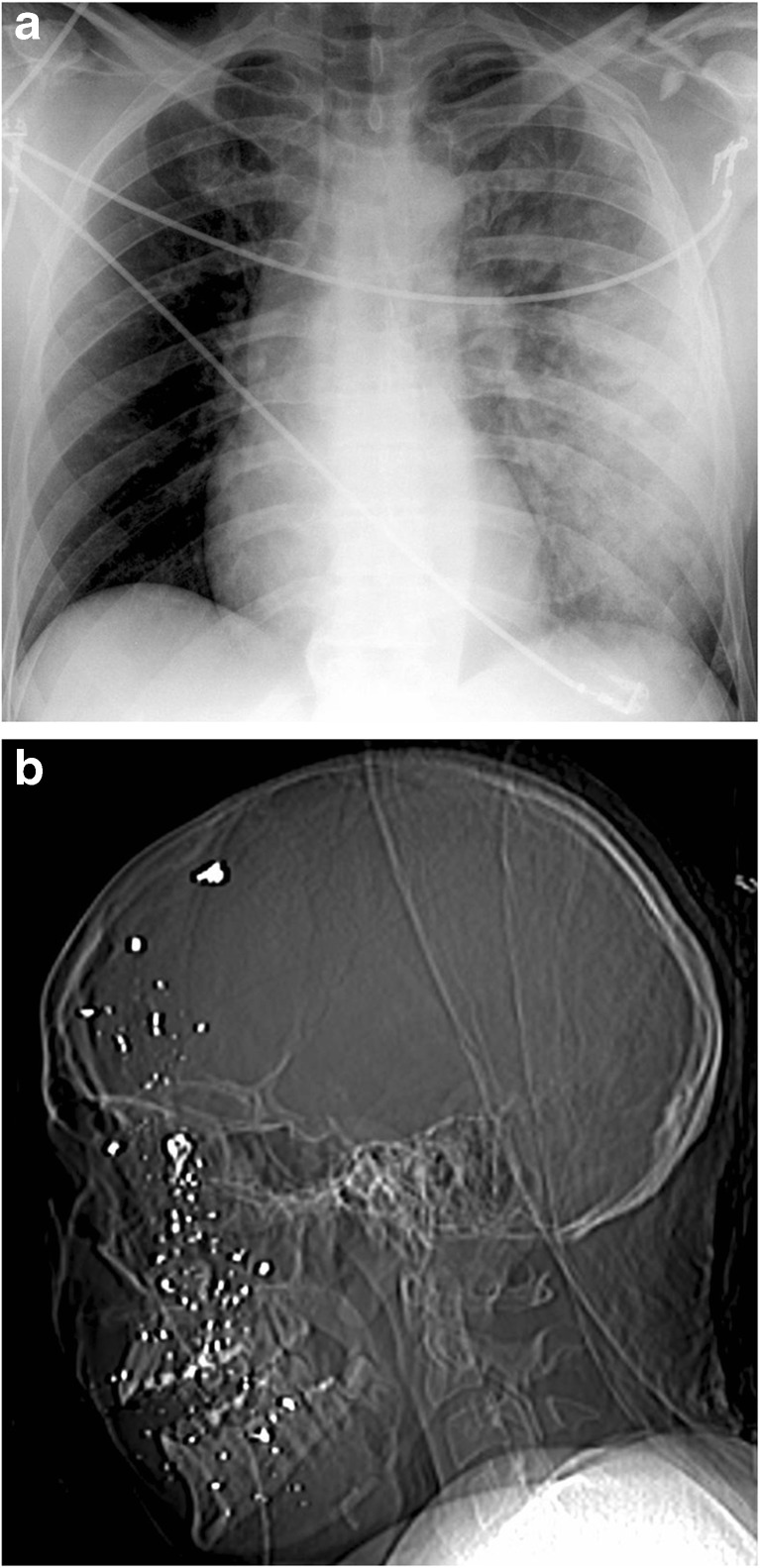
Imaging of two patients in the emergency department. **a** Plain film of the chest of a patient with blast injury to the upper left chest causing pulmonary contusion, comminuted fracture in the lateral part of the clavicle and multiple rib fractures. **b** CT scout of patient from the shooting incident with fragmenting injury to the face and head

The ten CT examinations performed in the ED included three head CTs and one WBCT in patients from the bomb explosion, and one torso CT, two head CTs, and three WBCTs in the gunshot patients (Fig. 2).

After discharge from the ED, one patient from the bomb explosion underwent a torso CT within 1 h after a previous head CT in the ED, another two with no previous CT examination underwent WBCT, and three had a follow-up head CT in the RD after 5–9 h.

Among the gunshot patients, three underwent WBCT, one had a head CT, and two had torso CT after discharge from the ED. In addition, three others had a follow-up head CT after 3–5 h.

All five CTs of the extremities were performed in combination with a WBCT, and all but one were performed in the RD.

Three CT scanners were running simultaneously during the period 18.00–19.00. The rest of the study time no more than two of four CT scanners were busy at the same time. No patients were referred to the angiography suites.

Imaging in non-trauma patients during the study period included eight chest x-rays—one with an additional pelvic x-ray—and two patients had a CT examination, all outside the ED.

In addition to the six radiographers scheduled on the afternoon shift (15.00–22.30) and two on the night shift (22.00–08.00), a further 19 radiographers worked simultaneously (Fig. **1**). Of 15 radiology consultants at work that day, ten stayed on after hours, and three more joined them.

There were up to six trauma teams in the ED, with two radiographers in each. The initial interpretation of all imaging was reported verbally to the surgical trauma team leader. Two registrars performed FAST in the major trauma bays. Two radiology consultants interpreted plain films in the ED. Following transfer of plain films to PACS, the exams were reread in the RD, and a written report made available in the RIS and patient chart system. The CT examinations in the ED were initially interpreted by one radiology registrar, and then reread and reported electronically by a radiology consultant in the RD. The CT examinations performed in the RD were interpreted and reported consecutively by the radiology consultants.

## Discussion

This study presents the initial radiology response in the regional trauma centre during the twin terror attacks in Norway on 22 July 2011. There was no shortage of radiology staff, and the great majority of the 24 included patients underwent diagnostic imaging in the ED.

Our findings confirm the importance of timely imaging in patients with potentially severe injuries. The MAC strategy in the ED, with limited use of imaging, emphasis on plain film examinations, and CT reserved for potentially severe head injuries, was successful in preventing the ED from becoming a bottleneck.

Trauma centre response during two recent bomb attacks with a similar number of severely wounded, i.e. London 2005 and Boston 2013, have been reported [2, 13]. In both events, the MAC strategy was used in the ED. As in the Boston report, we experienced queuing in front of the plate reader for plain films when the first victims had just arrived (personal observation, data not shown). However, this was not observed for the victims from the second attack, despite more frequent use of plain films in this group. The more frequent use of initial imaging in the gunshot patients than in those injured from the bomb explosion can be explained by the fact that the arrival of victims from the shooting incident was more spread out over time, with the overall situation becoming less unpredictable and the routines in the ED having had time to settle. This change of phases during an MCI has been reported by others [14]. However, our findings also indicate that plain films can be used more liberally in hospitals that have modern digital radiography units with wireless image transfer. This technology was acquired in our centre after 2011, and will likely eliminate the bottleneck from the plate reader queuing which was observed by us and others [13]. A previous prospective observational study from our centre included 104 trauma patients who underwent an early FAST performed by a radiologist in the ED. The authors reported a sensitivity and a specificity of 62% and 96%, respectively, and concluded that a negative FAST cannot reliably rule out intraabdominal bleeding in unstable patients [15]. The diagnostic uncertainty of FAST was also reflected in the current report with two laparotomies elicited by a positive FAST turned out negative. However, in spite of the recognised practical and diagnostic limitations, FAST as a screening tool in the ED may still be justified as this modality puts little demand on the radiology resources compared to CT [2, 15, 16]. During both the London and the Boston incidents, approximately 20% of the patients underwent CT examinations initially, compared to 75% in our report. The more frequent use of initial CT scanning after the twin attacks in Norway can be explained by the prehospital triage preventing most walking wounded from coming to the trauma centre; hence OUHU received only the more severely injured patients, as reflected in a higher mean ISS score in need of radiological imaging when capacity allowed. Half of the initial CT examinations were performed in the ED. More than half of these involved a non-head CT, and except for one gunshot patient who had an extremity CT and WBCT, the non-head CT examinations in the ED were of patients with more severe injuries (ISS range 20–59) arriving late from the two scenes. The MAC strategy defers all non-head CT examinations to a later stage outside the ED. One WBCT scan was performed in the ED after the bomb explosion. This is probably justifiable, since reliable information had confirmed that no more patients were expected from the scene. However, during the admission period after the shooting incident, four non-head CT scans were performed in the ED, potentially delaying patient flow. From Boston, it was reported that a high number of imaging referrals had to be considered and turned down by the radiologists, while in our setting, the surgical trauma team leader was in charge of the decision to perform diagnostic imaging. In order to avoid deviations from the MAC strategy with excessive use of scarce CT capacity in the ED in future MCIs, the disaster plan can dictate that the radiologist cancel all but head CTs until it is confirmed that no more patients are expected.

In addition, the mobilisation and volunteering of personnel resulted in at least a tripling of the radiology staff compared to the routine afternoon staffing. Similar mobilisation of radiology staff during MCIs has also been reported by others [4]. The mobilisation of hospital personnel is part of the red code activation, but the number of available staff will be influenced by factors unrelated to the hospital—in our case, the time coinciding with Friday afternoon shift overlap and July being the main summer vacation time in Norway. The redirection of all other emergency patients to other nearby hospitals relieved the overall capacity [5]. Although the workload on radiology staff might seem moderate for a large university hospital, the time-critical and unpredictable nature of MCIs challenges the logistics and capacity of the radiology services.

This study suffers from the weaknesses associated with its retrospective nature, and the busy nature of the initial phase of an MCI. However, recall bias is likely to be negligible in our study, as the results were retrievable from the hospital’s electronic files. However, an unbiased comparison between different strategies cannot be undertaken based on our observational data.

In conclusion, sufficient radiology resources were available in the regional trauma centre during the twin terrorist attacks in Norway in 2011. Following the initial assessment and discharge from the ED, the available plain film and CT capacity was never exceeded. Plain film capacity challenges have since been solved with new digital radiography units. A MAC strategy in the ED dictates CT examinations to be reserved for suspected severe head injuries, and the radiologist may in the future cancel all non-head CTs until no additional patients are expected.
